# Beyond PrEP: The Imperative for an HIV Vaccine to End the Epidemic in Africa

**DOI:** 10.3390/vaccines14070627

**Published:** 2026-07-17

**Authors:** Nicaise Ndembi, Morenike O. Folayan, Anthony Pilorget, Nyanda E. Ntinginya, Betty Mwesigwa, Trevor A. Crowell, Nyaradzo M. Mgodi, Jerome H. Kim

**Affiliations:** 1 International Vaccine Institute, Africa Regional Office, Nyarugenge, 1 KN 78 St, Kigali P.O. Box 3413, Rwanda; 2 Oral Health Initiative, Nigerian Institute of Medical Research, Yaba, Lagos 100001, Nigeria; 3 Department of Child Dental Health, Obafemi Awolowo University, Ile-Ife 22005, Nigeria; 4 Department of Public Health Dentistry, Saveetha Dental College and Hospitals, Saveetha Institute of Medical and Technical Sciences, Saveetha University, Chennai 600077, Tamil Nadu, India; 5 Mbeya Medical Research Centre, National Institute for Medical Research, Mbeya P.O. Box 2410, Tanzania; nyanda155@gmail.com; 6 Makerere University Walter Reed Program, Kampala P.O. Box 16524, Uganda; 7 U.S. Military HIV Research Program, Center for Infectious Disease Research, Walter Reed Army Institute of Research, Silver Spring, MD 20910, USA; 8 Henry M. Jackson Foundation for the Advancement of Military Medicine, Bethesda, MD 20817, USA; 9 Clinical Trials Research Centre, University of Zimbabwe, Harare P.O. Box MP167, Zimbabwe; 10 International Vaccine Institute, Seoul 08826, Republic of Korea

**Keywords:** HIV/AIDS, prevention, Africa, vaccines, pre-exposure prophylaxis

## Abstract

The era of highly effective pre-exposure prophylaxis (PrEP) has transformed HIV prevention, yet global HIV incidence remains unacceptably high, particularly in sub-Saharan Africa. While antiretroviral-based prevention is critical, persistent structural barriers to access and adherence highlight the urgent need for a complementary preventive HIV vaccine. In this narrative review and perspective, we argue that scientific progress toward an HIV vaccine must be matched by equally ambitious preparedness investments across three domains: manufacturing and supply chains, clinical trial and regulatory infrastructure, and delivery systems and community engagement. Drawing on lessons from the global rollout of PrEP, post-exposure prophylaxis (PEP), and the COVID-19 pandemic response, we outline a five-pillar strategic roadmap. This roadmap focuses on ensuring that a future HIV vaccine reaches the people who need it most from the moment of regulatory authorization, calling for co-investment in diverse platforms, binding advance market commitments, logistical simulation, and proactive planning for post-licensure effectiveness trials. Preparedness is not secondary to science; it is its essential partner.

## 1. Introduction

HIV pre-exposure prophylaxis (PrEP) is a highly effective biomedical intervention that has reshaped HIV prevention and become a critical component of global efforts to end the epidemic [1]. PrEP substantially reduces HIV incidence, particularly among populations disproportionately affected by HIV. However, despite the scale-up of PrEP and other interventions, global HIV incidence remains unacceptably high. In 2023, there were an estimated 1.3 million new HIV infections globally, with sub-Saharan Africa bearing the highest burden [2]. Current trends are not on track to achieve the UNAIDS target of a 90% reduction in new infections from 2010 levels [3].

While antiretroviral-based prevention tools are essential, they alone are unlikely to achieve sustained epidemic control in Africa without complementary strategies that address health system capacity constraints and social determinants. A safe and effective preventive vaccine remains a critical missing pillar in the comprehensive HIV prevention package. Vaccines are among the most scalable public health interventions, capable of providing durable protection without requiring continuous daily adherence. Recent advances in research rekindle hope for an HIV vaccine. Studies, especially on HIV-specific broadly neutralizing antibodies (bNAbs), which target diverse HIV strains, reveal promising pathways for harnessing the body’s own immune system to prevent HIV acquisition [4,5,6].

It is crucial to note that PrEP and vaccines are not interchangeable tools. A vaccine will not automatically solve the deep-rooted access, stigma, and health-system barriers that currently limit PrEP uptake. Rather, a vaccine will face its own unique logistical and delivery challenges. Therefore, the infrastructure, manufacturing, and delivery systems required to deploy an effective HIV vaccine need to be addressed proactively before such a vaccine has been developed. Preparedness is not secondary to science; it is its partner.

The purpose of this narrative review is to examine the preparedness gap in the HIV vaccine response. We assess infrastructure and partnership needs across manufacturing, regulatory systems, and delivery frameworks, and propose a strategic roadmap to ensure that a future HIV vaccine can be equitably deployed in Africa. This roadmap secures equitable access through global partnerships, tests distribution readiness, and integrates HIV vaccination with existing prevention strategies such as risk reduction counseling, condoms and condom-compatible lubricants, post-exposure prophylaxis (PEP), and PrEP [7].

## 2. Current Landscape of Pre-Exposure Prophylaxis (PrEP)

PrEP involves the use of antiretroviral drugs by HIV-negative individuals to protect against HIV acquisition. Daily oral PrEP, primarily co-formulated tenofovir disoproxil fumarate and emtricitabine (TDF/FTC), has been the cornerstone of PrEP programs and reduces HIV risk by >90% in adherent users. The recent approval of twice-yearly injectable lenacapavir for HIV prevention represents a landmark advance, offering a long-acting option that substantially reduces the daily adherence burden [8,9]. However, real-world effectiveness in sub-Saharan Africa is heavily hampered by access disparities and adherence challenges. Structural barriers, including stigma, healthcare inequity, and the logistical burden of frequent clinic visits, disproportionately impede PrEP uptake among vulnerable populations such as adolescent girls and young women (AGYW) and key populations [10,11].

Recent advances have introduced long-acting PrEP formulations, such as injectable cabotegravir (CAB-LA) administered every two months, and lenacapavir, administered twice yearly, which demonstrated remarkable efficacy in the PURPOSE 1 trial in Africa [8,12]. While these long-acting agents substantially reduce the daily adherence burden, they do not eliminate systemic obstacles such as clinic access, supply chain interruptions, and healthcare-associated stigma [13], particularly in East and Southern Africa, where the per capita burden of HIV is the highest [14]. There is indeed a complex interplay of social norms, perceived risks, and accessibility that influences PrEP’s effective implementation, especially among vulnerable populations [15]. Furthermore, equity risks are important in the current global environment, where access to novel agents has historically been concentrated in high-income settings before reaching the communities that bear the greatest burden of disease [16].

## 3. Post-Exposure Prophylaxis (PEP): Efficacy and Barriers

Post-exposure prophylaxis (PEP) is the use of a short course of antiretroviral drugs (usually 28 days) initiated within 72 h after a potential exposure to HIV. Decades of experience and systematic reviews have shown PEP to be highly effective in reducing HIV acquisition following occupational and non-occupational exposures [17,18]. Modern PEP regimens typically utilize well-tolerated integrase strand transfer inhibitors (INSTIs) [19].

Despite its efficacy and inclusion in WHO guidelines, PEP implementation in sub-Saharan Africa has been severely limited. It is often restricted to centralized facilities and cases of occupational exposure or sexual violence, resulting in missed opportunities for broader HIV prevention [18]. Barriers include a lack of knowledge among both providers and the public, the strict 72 h initiation window, and poor completion rates of the 28-day regimen [17]. Recent 2024 WHO guidelines advocate for decentralized, community-based distribution of PEP and task sharing to improve rapid access, highlighting the need for improved delivery models for all antiretroviral prevention tools [20].

## 4. The Biological Challenge of HIV Vaccine Development

An effective HIV vaccine offers several advantages in the African context. Vaccines are among the most scalable and cost-effective public health interventions, capable of reaching large populations through established immunization infrastructures. Unlike daily or episodic drug-based prevention strategies, vaccines can provide durable protection without requiring continuous adherence, thereby addressing a key limitation of PrEP programs. Moreover, a vaccine strategy can be integrated into existing preventive health platforms, including adolescent and adult immunization initiatives if delivered equitably.

Despite this, the development of an effective HIV vaccine remains uniquely challenging due to the virus’s biological complexity and immune evasion strategies. Indeed, HIV has an exceptionally high mutation rate and an extremely diverse envelope that facilitates rapid viral escape [21]. Antigenic variations and dense glycan shielding obscure conserved epitopes, reducing recognition and viral clearance by the host immune system [22]. Furthermore, HIV integrates into host genomes, establishing latent reservoirs resistant to immune clearance within days of initial infection [23]. This means a vaccine must achieve near-complete suppression of early viral replication to prevent reservoir establishment, a far more demanding immunological target than is required for most other pathogens. HIV induces immune dysregulation and CD4+ T-cell depletion that impair antiviral immunity [24,25]. An effective vaccine must elicit durable bNAbs and robust cellular immunity to overcome these challenges. Unlike influenza, where annual reformulations suffice, endogenous development of broadly neutralizing antibodies (bNAbs) against HIV requires years of maturation during chronic infection, a process that vaccine strategies must accelerate to outpace viral evolution [26].

Traditional attenuated or killed-pathogen vaccine approaches have failed to deliver a viable HIV vaccine. While current approaches based on mRNA and viral vector vaccines have enabled the induction of neutralizing antibodies with improved breadth, the levels achieved in vaccine recipients remain insufficient. Several current vaccine development strategies focus on immunogens that prime germline B cells and mimic sequential antigenic exposure without infection risk [27]. Memory B-cell studies reveal potential for cross-reactive protection [28], and identifying immunogenetic factors that predispose individuals to breadth will help tailor vaccines across populations. However, bNAbs often show reduced potency in vivo, necessitating physiologically relevant models such as those that use human peripheral blood mononuclear cells (PBMCs) for evaluation [29].

The innate immune system also shapes vaccine outcomes. Rationally designed adjuvants that bias Th1 responses enhance both antibody and cytotoxic T-cell responses [30,31]. Failures of advanced trials highlight the need for novel adjuvants, delivery systems, and immunogen engineering. Combining adjuvants with advanced platforms, including nanoparticles, biomaterials, and bioconjugates, are promising as they may offer better control over immune activation [32].

Next-generation approaches integrate computational epitope prediction with nanoparticle- and polymer-based carriers that protect antigens, improve targeting, and enable needle-free delivery [33,34]. mRNA vaccines, with proven scalability and adaptability, further expand possibilities [35]. Multi-epitope design, virus-like particles, and mucosal strategies remain central to countering HIV’s variability and mucosal transmission [36]. Critical scientific questions still remain regarding optimal immunization schedules, durability of responses, and clinical efficacy and effectiveness. However, even if an HIV vaccine offers durable, scalable protection, there are HIV vaccine preparedness gaps that need to be addressed.

## 5. Active Vaccines and Passive Immunization (bNAbs)

The scientific pursuit of HIV prevention currently involves both active vaccination platforms and passive immunization using monoclonal antibodies. Several advanced platforms are being utilized to elicit an active immune response against HIV. Table 1 summarizes these approaches.

### Passive Immunization with Broadly Neutralizing Antibodies (bNAbs)

While active vaccines aim to teach the body to make its own antibodies, passive immunization involves the direct administration of broadly neutralizing antibodies (bNAbs). As reviewed by Timofeeva et al. (2022), bNAbs are post-immune antibodies naturally found in a subset of patients with HIV that can neutralize a wide range of HIV-1 strains by targeting specific conserved viral epitopes [37].

Passive immunization with bNAbs (such as VRC01) has provided proof-of-concept that antibody-mediated prevention can protect against HIV acquisition in humans [4,5]. However, bNAbs are fundamentally different from active vaccines; they are biologic drugs that require specialized mammalian cell culture manufacturing, intravenous or subcutaneous administration, and have a limited half-life requiring repeated dosing. They serve both as a standalone prevention strategy and as a structural guide for designing active vaccines intended to elicit similar antibodies in vivo.

## 6. The Preparedness Gap: Manufacturing and Supply Chains

Leveraging adaptable vaccine platforms—mRNA, adenovirus vectors, or nanoparticles—can dramatically accelerate production timelines once a candidate is approved for clinical use. The unprecedented deployment of COVID-19 vaccines was achieved through the parallelization of development and manufacturing phases, funding large-scale production while Phase III trials were still underway [38].

HIV vaccine development is now compounded by the logistical complexities of modern candidates. An efficacious vaccination strategy will likely involve sophisticated regimens, such as heterologous prime-boosts combining different platforms (e.g., a viral vector prime followed by a protein or mRNA boost) or the delivery of stable formulations of bNAbs. Modern vaccine platforms and bNAbs often require stringent cold-chain storage (e.g., −20 °C to −70 °C for mRNA platforms), posing significant manufacturing and rollout challenges, particularly in resource-limited settings with unreliable electricity and transportation infrastructure [39]. Proactive engagement with global manufacturing partners is therefore a vital prerequisite that must be integrated into the clinical development pathway.

Rather than framing HIV primarily through the lens of an emerging or pandemic threat, the field should increasingly approach it as a persistent, high-burden infectious disease that is closely tied to poverty, inequality, and weak health system infrastructure. In that setting, preparedness should extend beyond investment in vaccine technologies alone and include the development of durable programs that integrate vaccination, PrEP, diagnostics, treatment, maternal and child health services, and community-based prevention. Continued investment in diverse vaccine platforms, including mRNA, viral vectors, and nanoparticle arrays, remains important given the exceptional biological challenges posed by HIV [40]. However, the ultimate goal is not simply to ensure rapid manufacturing once an effective immunogen is identified, but to embed new biomedical tools within equitable delivery systems capable of sustained production, distribution, access, and uptake in the populations most affected by the disease.

The early COVID-19 rollout starkly illustrated vaccine nationalism, where high-income countries secured the vast majority of doses, leaving Africa behind [41]. High-income countries (HICs), representing just 16% of the world’s population, secured an estimated 70% of the available vaccine doses in 2021, leading to profound and lethal inequity [42,43,44]. This “me-first” approach not only deprived vulnerable populations in LMICs of protection but also allowed for the unchecked transmission of the virus, creating fertile ground for the emergence of new variants that threatened the entire globe.

For HIV, proactive, equitable distribution must be engineered into the development process. This requires building regional manufacturing capacity in low- and middle-income countries (LMICs). The WHO mRNA vaccine technology transfer hub in South Africa, led by Afrigen Biologics, is a critical step toward sustainable regional production [45]. Furthermore, the African HIV epidemic is not uniform. In 2023, South Africa had an estimated 7.8 million people living with HIV, Mozambique 2.5 million, and Nigeria 2.0 million [2]. While countries like Eswatini and Rwanda have made remarkable progress toward the 95-95-95 targets, others lag behind [14]. Regional manufacturing and localized supply chains are necessary to respond to these specific national burdens efficiently.

Unlike COVID-19, which generated an immediate global emergency response and a powerful commercial market, HIV vaccine development now sits in the space of a market failure, where scientific risk is high, prior failures have reduced perceived probability of success, and sustained investment is therefore unlikely to come from industry alone. For that reason, governments and public-sector funders must carry much of the burden of supporting the long, high-risk research pathway required to identify an efficacious immunogen. Yet funding discovery is only part of the challenge: if an effective HIV vaccine does emerge, questions of who will manufacture it, where it will be produced, and who will control supply will become decisive. In this context, regional manufacturing capacity, vaccine sovereignty, and supply security, especially in Africa, are not peripheral concerns but central requirements for equitable access. The most important lesson from COVID-19, therefore, is not simply speed, but the need to build public, regional, and globally coordinated systems capable of carrying a scientifically difficult and commercially unattractive HIV vaccine from discovery through production and equitable deployment [46].

The scientific triumph of an efficacious HIV vaccine will be rendered a moral failure if it is not met with an equally sophisticated and pre-funded plan for global manufacturing and equitable rollout. The core challenge is that scaling up manufacturing from clinical-trial volumes to commercial, global-scale production is a massively capital-intensive and time-consuming process. It requires manufacturers to retrofit or build new facilities with specialized bioreactors and purification lines; secure long-term contracts for often scarce raw materials; hire and train a skilled technical workforce; and establish and validate rigorous quality control processes. No private company will make these multi-billion-dollar investments without a clear financial guarantee of a market. This is where the models pioneered by global health organizations, like CEPI or The Gavi Alliance, become essential.

CEPI emerged to address the “valley of death” between promising research and scalable production by investing early in platform technologies and at-risk manufacturing before efficacy is known [47]. Similarly, Gavi and its partners have used Advance Market Commitments and related purchase guarantees to create predictable demand and give manufacturers the confidence to invest in scale-up [48]. It is also important to recognize that barriers to manufacturer participation extend beyond financing alone. Procurement rules, uncertainty about guaranteed demand, and delayed purchasing commitments can all discourage long-term engagement, especially for products intended primarily as global public goods [49,50,51]. Addressing these structural barriers to industry participation should therefore be an explicit component of any advance commitment framework.

## 7. The Preparedness Gap: Clinical Trial and Regulatory Infrastructure

### 7.1. Strengthening LMIC Clinical Trial Networks

The pathway to developing an HIV vaccine requires rigorous, large-scale clinical trials, which are associated with substantial operational costs and complex procurement needs, particularly in the resource-limited settings where the disease burden is highest. Late-stage (Phase IIb/III) HIV vaccine efficacy trials are inherently expensive and logistically demanding, often requiring tens of thousands of participants and years of follow-up to demonstrate statistical significance.

In many developing countries, particularly in Africa, clinical trial sites often require extensive capacity building to meet international Good Clinical Practice (GCP) and Good Clinical Laboratory Practice (GCLP) standards. This includes the retrofitting of existing facilities or the construction of new infrastructure to house accredited laboratories capable of processing complex immunological assays. Procurement for these trials extends beyond the investigational vaccine product itself; it encompasses a vast array of supplies, including rapid diagnostic tests, antiretroviral therapy (ART) for participants who may acquire HIV during the trial, cold-chain storage equipment for sample preservation, and advanced laboratory reagents.

Furthermore, the management of these complex, multi-center trials frequently necessitates the engagement of Contract Research Organizations (CROs). While CROs provide essential expertise in trial monitoring, regulatory compliance, data management, and quality assurance, their involvement constitutes a significant portion of the overall clinical trial budget. In Africa, leveraging local or regional CROs has been proposed as a strategy to build sustainable research capacity and potentially reduce the exorbitant costs associated with multinational CROs, though challenges remain regarding the availability of highly trained personnel and robust regulatory frameworks [52].

Existing HIV clinical networks in Africa, such as the HIV Vaccine Trials Network (HVTN), International AIDS Vaccine Initiative (IAVI), U.S. Military HIV Research Program (MHRP) and BRILLIANT Consortium, represent priceless global assets [53]. Other clinical trial networks, not necessarily focused on HIV vaccines, such as the Advancing Clinical Therapeutics Globally for HIV/AIDS and Other Infections (ACTG), HIV Prevention Trials Network (HPTN), and European and Developing Countries Clinical Trials Partnership (EDCTP) Networks of Excellence, can also be leveraged for vaccine testing through capacity augmentation. Strengthening these networks ensures rapid recruitment and generates efficacy data directly applicable to the populations most burdened by the epidemic, accounting for local genetic diversity and co-infections [54]. In addition, it is important to ensure that HIV vaccine research and development have real-world relevance. Trials conducted within the communities most burdened by the epidemic generate data on efficacy that are directly applicable to target populations. This includes understanding how co-infections (e.g., tuberculosis, malaria), genetic diversity, and different HIV subtypes might impact vaccine effectiveness.

### 7.2. Harmonizing Regulatory Pathways

The regulatory landscape for vaccines remains notoriously complex and fragmented, especially in Africa. A vaccine approved in one region can face months or even years of delay while navigating the distinct regulatory requirements of another, creating unacceptable inequities in access. Capacity enhancement of regulatory bodies and proactive harmonization is therefore essential to ensure the timely and equitable availability of vaccines worldwide.

Efforts such as those led by CEPI, AVAREF and AMA are advancing models of regulatory reliance and harmonization on the continent [55,56]. These include mechanisms such as joint reviews, reliance mechanisms, in which regulators accept approvals granted by stringent agencies such as the FDA or EMA or by regional bodies; and the adoption of common technical dossiers, which align submission requirements and ease the administrative burden on developers. For an HIV vaccine, efficient regulatory pathways must be predefined and agreed upon by global regulators. Once a candidate meets primary efficacy endpoints, data can be rapidly reviewed and approved for use, even as full licensure data continue to be collected. The true potential, however, lies in the synergy between strong trial networks and harmonized regulation. Data generated from robust, LMIC-led trials can be consolidated into a common dossier and evaluated through collaborative mechanisms, enabling faster and more equitable approvals across regions.

## 8. The Preparedness Gap: Delivery Systems and Community Engagement

Scientific triumph in the lab means little without sustainable, equitable and effective delivery systems on the ground. The history of global health is replete with examples of powerful technologies that failed to reach those most in need due to logistical neglect and a lack of community trust. For a future HIV vaccine, especially one with complex storage and dosing requirements, addressing the delivery and engagement gap is as urgent as the scientific research itself.

Many leading HIV vaccine candidates present substantial logistical hurdles. mRNA platforms, while rapidly producible, require ultra-cold storage (−20 °C to −70 °C), a capability absent in many primary health clinics in high-HIV-burden regions [39]. The manufacturing and distribution of such vaccines adds further complexity: in addition to the cold-chain infrastructure distinct from that used for traditional vaccines, kept at +4 °C, the cost and logistics of these new vaccines must be factored into preparedness planning from the outset. Targeted investments in solar-powered cold-chain equipment and formulation innovations (e.g., lyophilization) must be made before a vaccine is available.

An equitable rollout must be human-centered and co-designed with the community. Successful HIV programs have consistently shown that services delivered through community-based organizations have far higher uptake. These organizations, led by and for the affected populations, provide a bridge of trust and cultural competency. Funding and integrating these networks into vaccine rollout planning, from trial design to messaging and delivery, is non-negotiable [57]. This partnership is also strategic for proactively addressing vaccine hesitancy, which will be a significant challenge, fueled by decades of stigma and misinformation [58,59,60,61]. Counter-narratives must be led by trusted, authentic community influencers, not distant government officials. Investing in community-led communication campaigns now, before a vaccine is even available, builds a foundation of accurate information and readiness.

## 9. Strategic Roadmap for Success

The path from a successful HIV vaccine candidate to the end of the global epidemic is a complex journey riddled with operational, financial, and ethical hurdles. To navigate this successfully, the global health community must learn from past chapters in vaccinology, most notably the COVID-19 pandemic, with both its achievements and its failures, and draft a proactive plan. This strategy ensures that a scientific breakthrough is met not with logistical chaos, but with a coordinated, equitable, and efficient global response.

**Figure 1:**
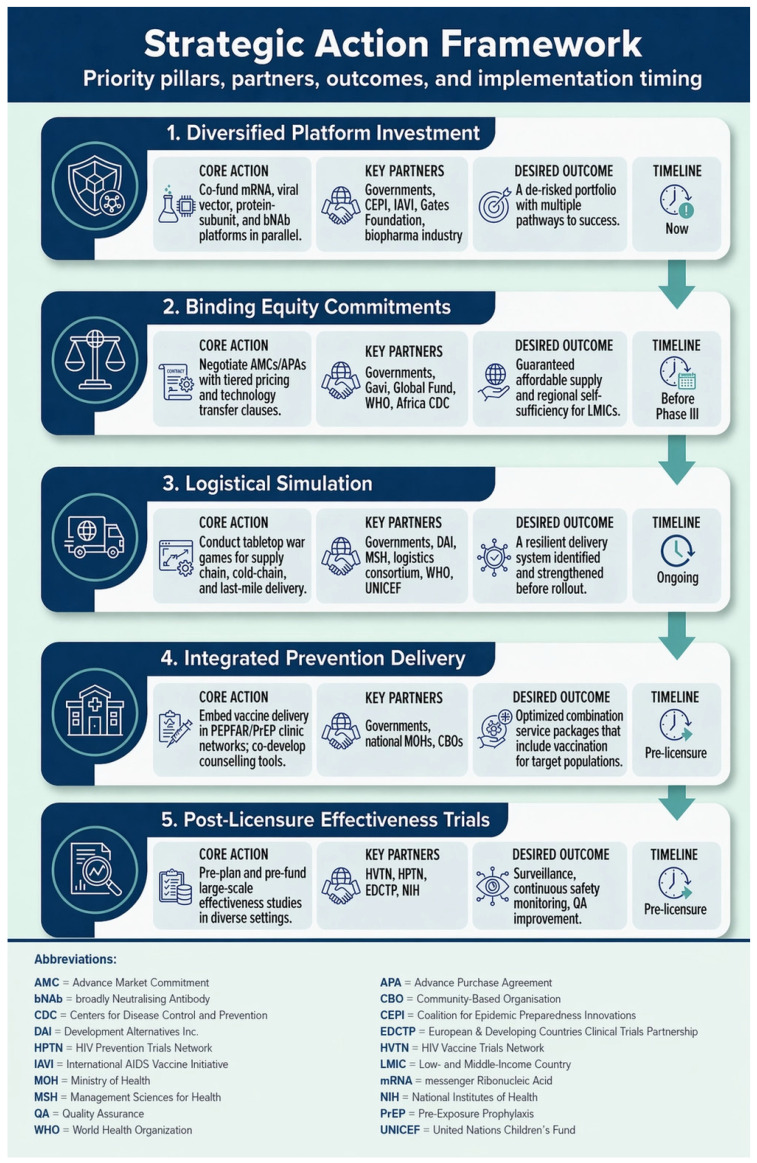
A Five-Pillar Roadmap for HIV Vaccine Preparedness.

The roadmap rests on five interconnected pillars: diversified investment, a binding commitment to equity, rigorous logistical simulation, seamless integration with existing prevention tools and the development of post-licensure effectiveness studies.(Figure 1).

**Pillar 1: Co-Investing in a Diverse Portfolio of Vaccine Platforms.** Given the formidable scientific challenges posed by HIV, concentrating resources on a single technological approach represents an unacceptable risk. A more robust strategy entails parallel funding for mRNA, viral vector, protein-subunit, and passive bNAb platforms. This “no regrets” investment model mitigates the risk of failure by ensuring that if one platform encounters scientific, safety, or manufacturing obstacles, others can advance [40]. Such a multi-platform strategy ensures that the entire field advances, even if only one candidate ultimately proves effective.

**Pillar 2: Pre-Committing to Equity Through Binding Financial and Contractual Agreements.** The world must move beyond rhetoric and pre-commit to equity through binding financial and contractual agreements. Entities like Gavi and The Global Fund must establish Advance Market Commitments that guarantee a viable market and a fair price for manufacturers, incentivizing them to scale up production in advance [48]. These agreements must include tiered pricing and clauses that prioritize dose allocation to high-incidence countries [49,50]. Concurrently, funding must be earmarked for technology transfer and capacity building within LMICs, exemplified by the WHO mRNA vaccine technology transfer hub in South Africa [45]. Programs like EDCTP and the Africa CDC’s Platform for Harmonized African Health Products Manufacturing, are essential to foster regional self-sufficiency and shorten supply chains for the continent most burdened by the epidemic [62].

**Pillar 3: Stress-Testing the System Through Rigorous Logistical Simulation.** The unique complexities of a potential HIV vaccine regimen, which may involve multiple doses, stringent cold-chain requirements, or specialized healthcare worker training, demand that we simulate the entire rollout through rigorous “war games”. The value of such proactive modeling has already been demonstrated by logistics consortia such as the USAID Global Health Supply Chain Program and those organized by Management Sciences for Health (MSH) and Development Alternatives inc. (DAI).

**Pillar 4: Designing for Synergistic Prevention from the Outset.** An HIV vaccine must be integrated thoughtfully into the existing landscape of prevention tools rather than introduced in a vacuum. Its introduction must be planned alongside highly effective PrEP to create a robust, person-centered prevention toolkit [8,13,63]. Operational research must begin immediately to evaluate combined regimens in clinical trials, develop integrated service delivery models for clinics, and determine optimal counseling for individuals offered both tools. Crucially, the vast and trusted PEPFAR-supported network of clinics, community advocates, and testing facilities represents the ideal channel for future vaccine distribution.

**Pillar 5: Planning and Financing Post-Licensure Effectiveness Trials.** The experience with the RTS,S malaria vaccine is instructive: even after regulatory approval, large-scale post-licensure effectiveness trials were required to generate the real-world evidence needed to inform national immunization program decisions, at an additional cost estimated at $40–50 million [64]. A similar trajectory is plausible for an HIV vaccine, particularly given the diversity of HIV subtypes, the heterogeneity of epidemic contexts across sub-Saharan Africa, South America, and Southeast Asia, and the likelihood that vaccine effectiveness may differ across populations. Pre-planning and pre-funding these effectiveness studies, including defining the trial networks, endpoints, and financing mechanisms before licensure, is therefore an essential component of the preparedness roadmap.

## 10. Conclusions

PrEP and PEP represent milestones rather than endpoints in HIV prevention. Their persistent implementation challenges underscore the urgent need for an HIV vaccine. However, a vaccine will not automatically resolve structural inequities. Scientific breakthroughs must be matched with strong manufacturing partnerships, agile regulatory processes, and community-integrated delivery models. Building the infrastructure for HIV vaccine delivery now, particularly across Africa, is essential to bridge the gap between scientific discovery and true epidemic control.

## Figures and Tables

**Table 1:** Summary of Current HIV Vaccine Platforms and Approaches.

Platform Type	Mechanism	Current Status/Challenges
mRNA Vaccines	Encodes HIV antigens (e.g., Env trimers) inside lipid nanoparticles to induce cellular production of immunogens.	Early clinical trials. Highly adaptable and scalable, but requires ultra-cold chain storage.
Viral Vectors	Uses harmless viruses (e.g., Adenovirus, Canarypox) to deliver HIV genetic material.	Clinical trials (e.g., ALVAC). Challenges with pre-existing immunity to the vector and durability of response.
Protein Subunit	Direct injection of engineered HIV proteins (e.g., stabilized Env trimers) with adjuvants.	Clinical trials. Difficult to manufacture stabilized trimers that accurately mimic the native viral spike.
Mucosal Vaccines	Targets mucosal immune system (IgA) at the primary sites of HIV entry.	Preclinical/Early clinical. Challenges in overcoming mucosal tolerance and achieving robust immunogenicity.

## Data Availability

No new data were created or analyzed in this study. Data sharing is not applicable to this article.
